# When Two Is Better Than One: Elements of Intravital Microscopy

**DOI:** 10.1371/journal.pbio.0030207

**Published:** 2005-06-14

**Authors:** David W Piston

## Abstract

What are the technical underpinnings of two-photon microscopy? What are the advantages of using two-photon microscopy versus conventional confocal microscopy?

Over the last 20 years, many cell biological studies have moved from the single-cell level to the tissue level, and even to whole animals. This progress has been led by developments in fluorescence microscopy that permit molecular observations from single cells within intact tissue or animals. Concurrent developments in fluorescent probes, especially the cloning of the Green Fluorescent Protein and its use in transgenic animals, have also fueled this movement. The key instrumental technology for this work is optical sectioning microscopy; in this technique, instead of fixing and physically sectioning a sample, the investigator obtains a 3-D dataset from an intact (and more importantly, live) specimen. The most common optical sectioning technique is confocal microscopy, where fluorescence is created throughout the sample and a confocal pinhole is placed in front of the detector so that only the in-focus fluorescence is recorded. For live samples, whose cells can be killed by the excitation light (via photo-toxicity, particularly of ultraviolet and blue wavelengths), confocal microscopy may not be an option. A more recently developed optical sectioning method is two-photon excitation microscopy (which also goes by the names multi-photon microscopy and nonlinear optical microscopy). As described below, two-photon excitation offers very significant advantages for the high-resolution imaging of thick living samples (as deep as 1 mm). Most importantly, two-photon imaging is now ready for prime time because of instrumental advances that have made it as easy to use as any other fluorescence microscopy technique.

## Fluorescence Excitation

To understand two-photon excitation and its advantages for imaging, it is helpful to understand a little bit about fluorescence. Fluorescence is the process of absorption and re-emission of light. Normally, a single light particle (photon) is absorbed by a fluorescent molecule, causing an excited state, which subsequently relaxes by emitting another photon. The excitation light is typically ultraviolet, blue, or green. Any time a photon that has the correct energy to cause the excited state comes in close contact with a fluorescent molecule, it may be absorbed. In contrast, two-photon excitation of fluorescence depends on the simultaneous absorption of two photons (each of which contains half the energy, typically red or infrared, needed to cause the excited state). For this simultaneous absorption to happen, the photons must be so crowded that there is a good chance two photons will simultaneously be at the same place as the fluorescent molecule. In a two-photon excitation microscope, the photons are crowded in both time and space. The photons are crowded in time through the use of short pulses of light, which are about 100 femtoseconds (one tenth of one millionth of one millionth of a second) in duration. This causes about a million times more photons to be present at the same time than would be present in a normal constant wave laser of the type commonly used in confocal microscopes. The photons are crowded in space by focusing through the microscope objective lens. As a single laser beam is focused in the microscope, the photons become more than a million times more crowded still. The combination of short pulses and focusing crowds the photons by a factor of over one trillion.

Even with the high powers used, the only place that photons become crowded enough that two of them would be interacting with a single fluorescent molecule at the same time is in a small region at the focus of the microscope. This region, called the focal volume, is the only place that two-photon excitation occurs. This localization of two-photon excitation leads to the advantages for deep-tissue imaging. If standard fluorescence microscopy is like probing the contents of a house by shining a powerful spotlight into the house from outside, two-photon excitation is more like taking a flashlight around the inside of the house; all of the excitation is generated inside the sample.

## High Resolution, High Contrast

**Figure 1:**
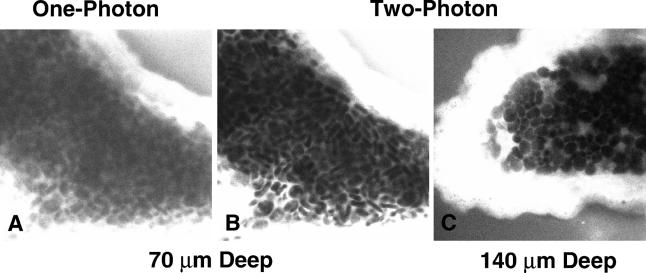
Images of a Shark Choroid Plexus Stained with Fluorescein (A) and (B) were collected 70 µm into the sample, and (C) was collected 140 µm into the sample. The contrast of the confocal image (A) is significantly degraded at this depth, while two-photon excitation at the same focal plane (B) allows the collection of an image with excellent intensity contrast. Further, using two-photon excitation to image deeper into the sample (C) does not significantly degrade the image contrast.

As stated above, the major advantage of two-photon excitation is its ability to permit high-resolution and high-contrast imaging from deep within intact living tissue. Figure 1 shows an intact shark choroid plexus that has been stained with fluorescein in the extracellular space. Figure 1A shows a confocal image taken 70 µm into the sample, which exhibits minimal contrast between the bright cell borders and the dark intracellular spaces. Figure 1B shows the same section acquired with two-photon excitation; the contrast is much greater. In fact, even with a much deeper section (140 µm into the sample) (Figure 1C), two-photon excitation still provides similar contrast.

One question that is often asked is, how deep can this approach go? The answer, of course, depends on the specific type of tissue, but a good rule of thumb is that one can image 6-fold deeper with two-photon excitation than with confocal microscopy. There are two reasons for this deeper penetration. The first is that there is no out-of-focus absorption in a two-photon excitation microscope. Because the photons are only crowded enough for two-photon excitation at the microscope focus, they are not absorbed by fluorescent molecules as they pass through the sample. In confocal microscopy, the excitation photons can be absorbed anywhere in the sample. Thus, a higher percentage of excitation photons reach the focus in two-photon excitation, and this advantage grows as the focus moves deeper into the sample. Greater excitation leads to greater signal, and in turn to increased contrast in the image.

**Figure 2:**
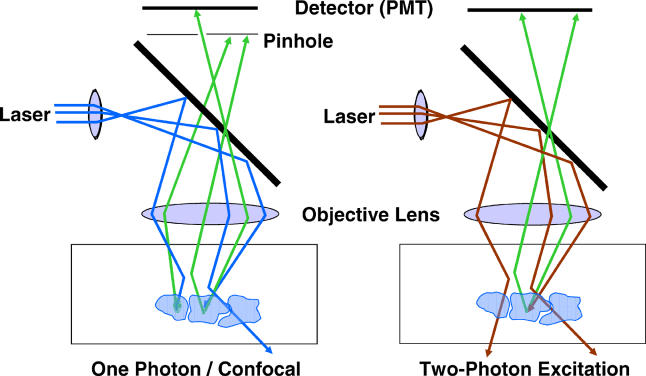
Effect of Scattering in Confocal Microscopy and Two-Photon Excitation Microscopy In confocal microscopy (shown on the left), blue excitation light reaches the focus, and green fluorescence from the focus is collected and passes through a pinhole. Scattering of the fluorescence causes it not to pass through the pinhole, thus reducing signal, while any scattering of the excitation beam can cause fluorescence, which adds background haze to the image. In two-photon excitation microscopy (shown on the right), because no pinhole is needed, the scattered fluorescence photons can still be collected, thus increasing the collected signal. Further, the scattering of a single red excitation photon does not cause background (and the chance of two photons scattering to the same place at the same time is zero).

The second reason for better depth penetration is that two-photon excitation imaging is less sensitive to scattering in the sample (Figure 2). This concept has not been well understood, and has been incorrectly reported in many papers. It is often stated that because the red and infrared photons used in two-photon excitation are less scattered by tissue, these photons can penetrate more deeply. While it is true that the photons are scattered slightly less than blue or green photons, this difference is small compared to the differences in sensitivity to scattering between confocal and two-photon excitation microscopy. In confocal microscopy, excitation photons that are scattered in the sample can cause fluorescence anywhere in the sample. As the laser power is increased in an attempt to image deeper into the sample, fluorescence due to scattered excitation also increases. This leads to a background haze in the image that reduces contrast.

The emitted fluorescence photons can also be scattered as they come out of the sample. When a fluorescent photon is scattered, it will not pass through the confocal pinhole, and therefore, will not be detected. This lowers the signal, which in turn lowers the image contrast. Thus, both the scattering of excitation light and emitted fluorescence lead to decreased contrast in the confocal image. For two-photon excitation, neither scattering event is deleterious to the image. As for scattering of the excitation photons, there is really no chance of two photons scattering to the same place at the same time, so even in a highly scattering sample, it is possible to increase the excitation power without generating background haze. As for the emitted photons, a two-photon excitation microscope collects most of the scattered fluorescence, since there is no pinhole needed (the only place fluorescence is being generated is in the focal spot). These two reasons, combined, allow two-photon excitation imaging to provide high contrast images from deep within intact tissue, although limitations in available laser power usually limit the depth penetration to less than 1 mm into the tissue. Further details about the advantages of two-photon excitation imaging are presented elsewhere [1–3].

## Looking Deeper

The advantages of two-photon excitation microscopy are truly realized for deep tissue imaging. While the technique can be used to image thinner samples, such as single cells, it will generally not be better than using confocal or deconvolution microscopy. In fact, these other approaches are better suited to such thin samples and also offer better spatial resolution. Further, there may be additional problems associated with two-photon excitation because of the extreme crowding of photons needed. With these high intensities, it is possible to activate other nonlinear processes, which can lead to increased photobleaching and photodamage, possibly negating the advantages of two-photon excitation in thinner samples.

As one might expect for such a complicated physical phenomenon, it was some time before two-photon excitation found its way into biological research. In fact, two-photon excitation was first predicted theoretically by Maria Goppert-Mayer in her 1931 PhD thesis at the University of Göttingen (Göttingen, Germany) [4], and was experimentally verified in a very early laser experiment by Kaiser and Garrett in 1961 [5]. It was not until the invention of powerful, ultrafast lasers that Denk et al. were able to bring two-photon into use for microscopy in 1990 [6]. Since that time, there has been considerable interest, and most major research institutions have made some effort to set up a two-photon excitation microscope. Despite the inherent advantages, though, two-photon excitation microscopes are sitting idle in many of these labs. There are a couple of reasons for this. First, the Ti:Sapphire lasers that have been available over the last 15 years are reliable and “hands-free” from a laser-jock perspective, but it has proven difficult for a typical biology lab to keep the lasers in optimal working condition. Second, many investigators did not have projects that were well-suited to the strengths of two-photon excitation microscopy. In these cases, the results were often no better than confocal microscopy, and thus the extra overhead to maintain the Ti:Sapphire laser was not well-justified.

These days, neither of these problems applies. The newest available lasers are in a single box, fully hands-off, and computer controlled. This permits any researcher to use two-photon excitation. Further, problems that are well-suited to the application of two-photon excitation have finally found the use of this powerful approach. For example, as demonstrated by two papers in this issue [7,8], researchers are now able to characterize the activities and motion of individual lymphocytes in intact lymph node [7] and thymus [8], making direct observations of phenomena that had only been inferred using other approaches. Coupled with the now-mature instrumentation, we should expect two-photon excitation imaging to play a key role in our future understanding of in vivo biological processes.
